# Supplementary material for: Haloarchaea swim slowly for optimal chemotactic efficiency in low nutrient environments
Source: Nat Commun. 2020 Sep 8;11:4453. doi: 10.1038/s41467-020-18253-7 (PMC7478972; doi:10.1038/s41467-020-18253-7)
Supplement: Supplementary file 1 — Supplementary Information [file 41467_2020_18253_MOESM1_ESM.pdf]

Supplementary Information

Haloarchaea Swim Slowly for Optimal Chemotactic Efficiency in Low Nutrient Environments

Katie L. Thornton[1], Jaimi K. Butler[2], Seth J. Davis[3,4], Bonnie K. Baxter[2] & Laurence G. Wilson*[1]

1. Department of Physics, University of York, Heslington, York, YO10 5DD, UK
2. Great Salt Lake Institute, Westminster College, 1840 South 1300 East, Salt Lake City, UT 84105, USA
3. Department of Biology, University of York, Heslington, York, YO10 5DD, UK
4. State Key Laboratory of Crop Stress Biology, School of Life Sciences, Henan University, Kaifeng 475004, China

* Corresponding author: laurence.wilson@york.ac.uk

Supplementary Figure 1: Cell morphology

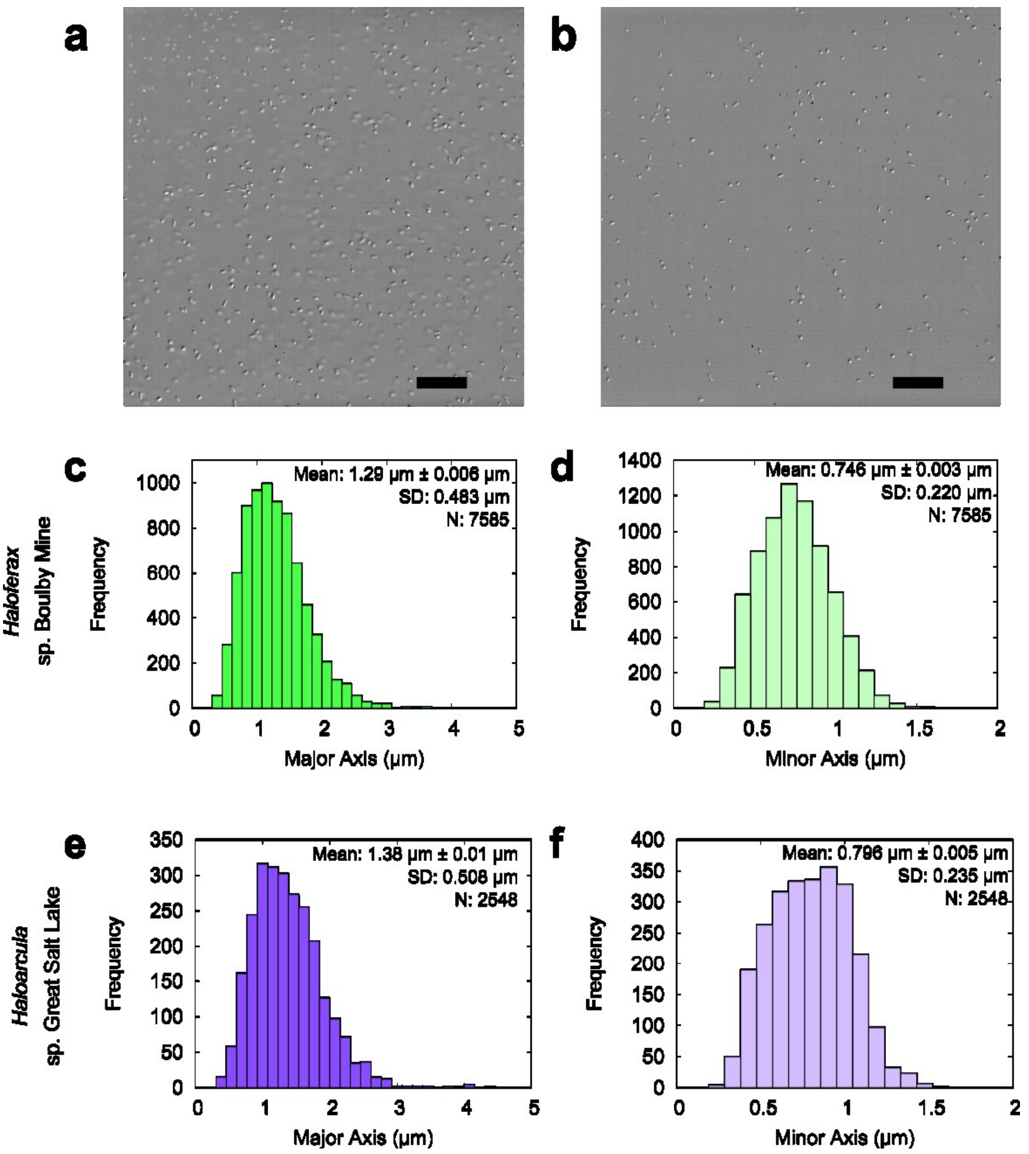

Supplementary Figure 1: Images of halophilic archaeal isolates. **a** *Haloferax* sp. Boulby Mine (HXBM). **b** *Haloarcula* sp. Great Salt Lake (HGSL). The images were obtained by differential interference contrast microscopy at an optical magnification of 90× (60× oil immersion lens and a 1.5× tube lens) with numerical aperture of 1.45. Scale bars represent 20 µm. A threshold was applied to the images, converting them to binary arrays highlighting the cells. These binary images were sized using a routine written in LabVIEW, ignoring any objects with an area of less than 6 pixels. Ellipses were fitted to the objects identified, and the major and minor axes of the ellipses are shown in the following panels **c** Distribution of major axis length of ellipses fitted to binarized images of HXBM. **d** Minor axis length for ellipses fitted to cell images of HXBM. **e,f** Major and minor fitted ellipse axis lengths for HGSL. The mean ± s.e.m. (standard error of the mean), standard deviation (SD) and number of cell images per strain are indicated in each panel.

Supplementary Figure 2: Location of reversal events

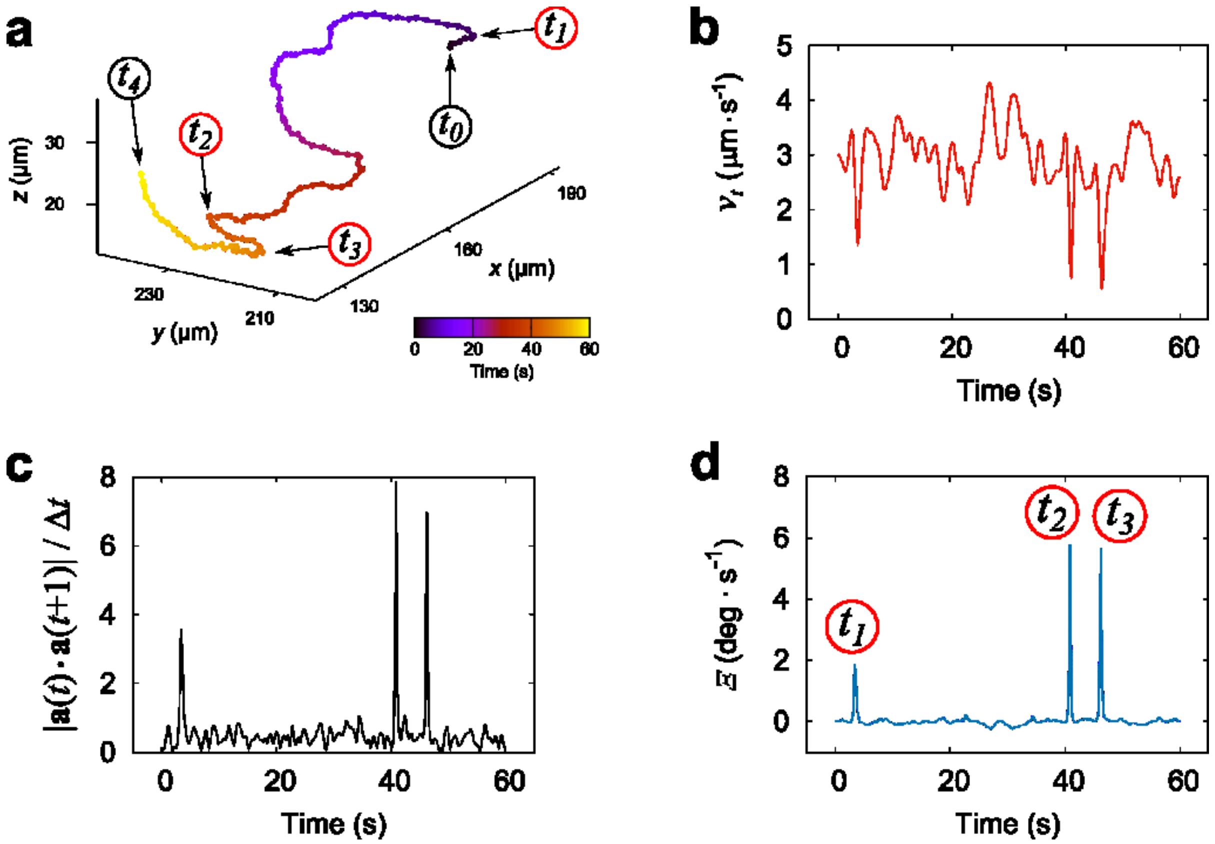

Supplementary Figure 2. a An example track from the strain *Haloarcula* sp. Great Salt Lake (HGSL), with events of interest highlighted. The points at t_0 and t_4 correspond to the start and end of the track, respectively. Points t_1, t_2, t_3 are instances where the cell has reversed swimming direction. **b** The instantaneous swimming speed of the cell, calculated form the spline-smoothed trajectories (see Methods). Reversal events can be seen as dips in swimming speed. **c** The instantaneous change in direction exhibited by the swimming cell. Here, the reversal events are observed as peaks. **d** The heuristic metric Ξ (see Methods), with reversal events identified.

Supplementary Figure 3: Swimming in saltwater

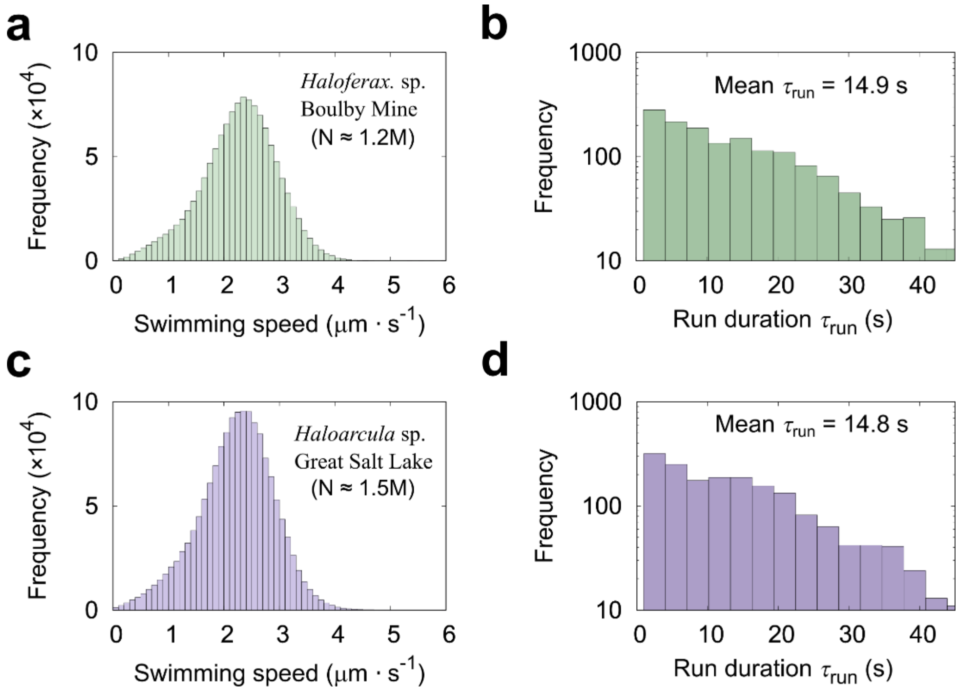

Supplementary Figure 3. Run/reverse behaviour of archaeal strains swimming in saltwater media. Experiments to verify chemotactic performance were conducted in gradients of methionine superimposed on a 'bare' saltwater medium devoid of nutrients, so we performed additional controls to ensure that the swimming behaviour was consistent both with and without nutrient supplement. Cells were grown as described in the methods section of this paper, and then washed three times into 25% w/v saltwater media [1]. The cells were observed using our digital holographic microscope and their run/reverse behaviour analysed. The distribution of swimming speeds is very similar to that observed in the presence of nutrients. The distribution of run durations in strain *Haloferax* sp. Boulby Mine (HXBM) is very similar to that observed in nutrient media, while cells of strain *Haloarcula* sp. Great Salt Lake (HGSL) display a mean run duration in saltwater that is longer than that in nutrient media, albeit only by around 20% (14.8 s, compared to 12.1 s in nutrient media).
a Distribution of swimming speeds obtained from HXBM, with a mean swimming speed of 2.3 μm s^{-1} ± 0.6 μm s^{-1} (mean ± s.d.). **b** Distribution of run durations for HXBM in saltwater. The mean run duration is very close to that obtained in nutrient media. **c** Distribution of swimming speeds for (HGSL), with a mean swimming speed of 2.2 μm s^{-1} ± 0.6 μm s^{-1} (mean ± s.d.). **d** Distribution of run durations for HGSL. This species exhibits slightly longer runs in salt water (approx. 20% longer), compared with results from nutrient media.

Supplementary Figure 4: Spatial variation of swimming speed

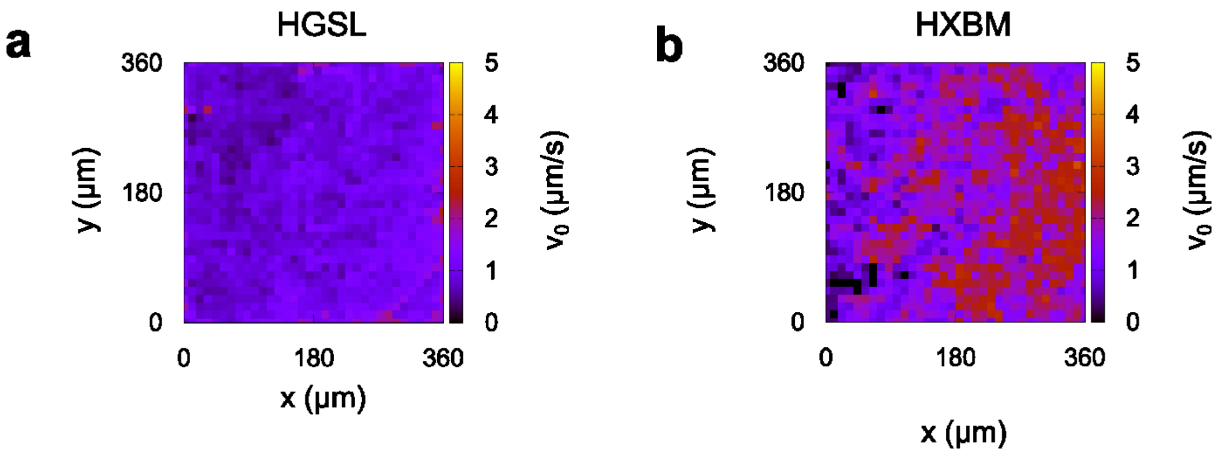

Supplementary Figure 4. In the experimental part of our study, we anticipated that there might be a metabolic effect on the archaeal strains due to the presence of the chemical gradient. To test this, we binned the swimming speeds of cells within the gradient as a function of position within the field of view. The results for strain *Haloarcula* sp. Great Salt Lake (HGSL), are shown in panel **a**, and those for *Haloferax* sp. Boulby Mine (HXBM) are shown in panel **b**. All bins measure 10×10 μm^2, and bins in which no cells were observed are coloured black. Both strains show a more or less uniform distribution of swimming speeds throughout the field of view. This suggests that although there could be a slight metabolic effect, perhaps accounting for the slightly longer average run duration in HGSL compared to the gradient-free case, we do not observe significant chemokinetic effects [2]. This strengthens the case for using our simplified chemotactic model in the simulations.

Supplementary Figure 5: Effect of varying chemotactic sensitivity W

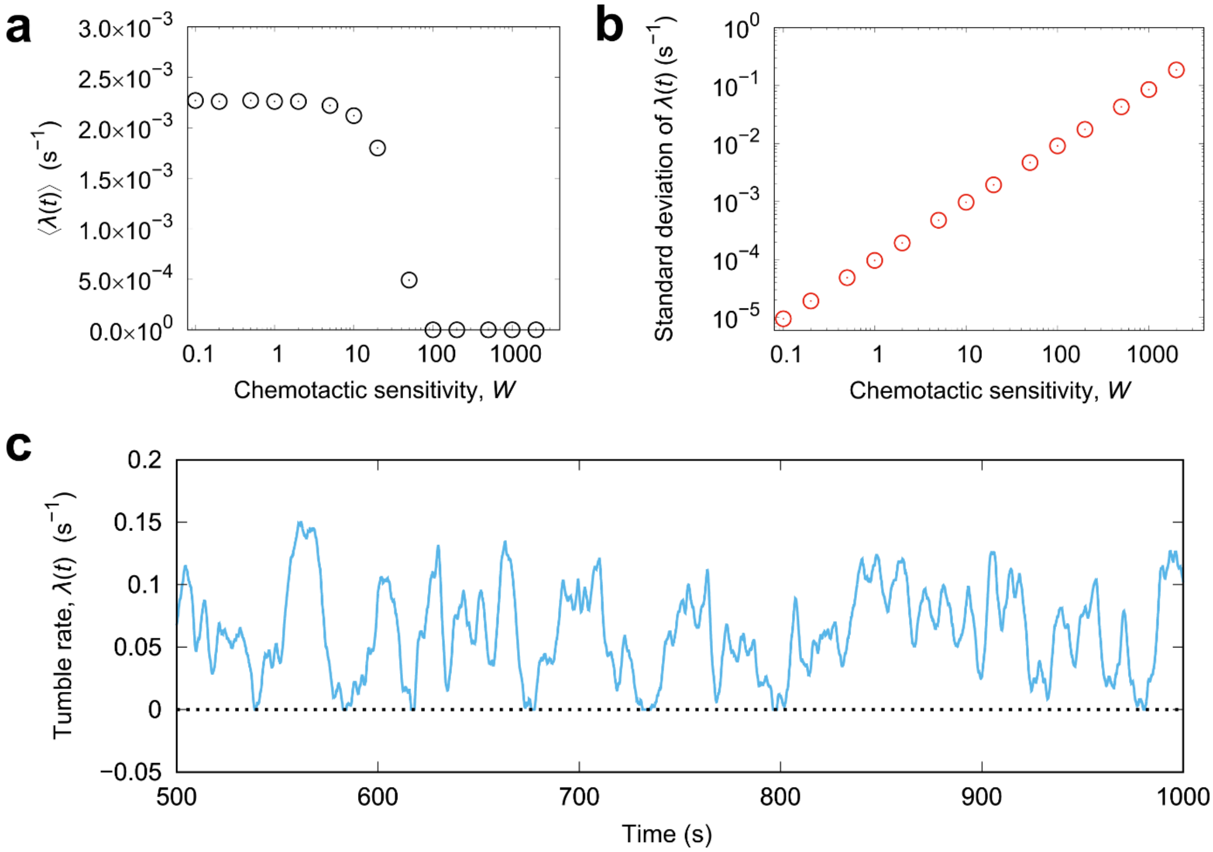

Supplementary Figure 5. Effect of varying chemotactic sensitivity W. **a** The average reversal rate $\langle\lambda(t)\rangle$ as a function of W, showing a reversal rate of zero (saturation) above *W*=50. The physical interpretation of this behaviour is a cell that very rarely reverses, and we map any value of $\lambda(t) < 0$ to zero. Error bars have been omitted as they are smaller than the data points. **b** Standard deviation in reversal rate $\lambda(t)$ as a function of W. The standard deviation increases linearly with W. Error bars have been omitted as they are smaller than the data points. **c** An example series showing reversal rate as a function of time for a cell swimming at 2 μm s^{-1}. The dotted black line shows the point at which receptors become saturated and the reversal rate drops to zero. As a result of these experiments, we chose *W* = 20 for our main simulations: the range of the reversal rate is maximised, while the saturation of receptors, i.e. duration for which $\lambda(t)$ = 0, is minimised. Our relatively high sensitivity (*i.e.* range of $\lambda(t)$) is therefore still consistent with a linear chemotactic detection system that can be described with a convolution. Although some components of the signal transduction system (e.g. the response regulator CheY) are conserved between eubacteria and archaea, some components are distinct, such as the archaeal-specific adapter protein CheF. We are only beginning to understand the details of how these domain-specific components function. Many of the best-known eubacterial signal transduction systems modulate their response by (e.g.) methylation of receptors or by the dephosphorylation of CheY (CheZ performs this role in *E. coli*, for example). Moreover, receptors in eubacteria

are clustered to allow for cooperative binding events as neighbouring receptors interact. Any of these modulation approaches could be present in archaea but may not use eubacterial components and could well give rise to a high sensitivity. This seems especially likely in species that live in nutrient-poor environments where the ability to detect weak, ephemeral gradients would be an advantage.

Supplementary Figure 6: Three-dimensional *E. coli* tracking

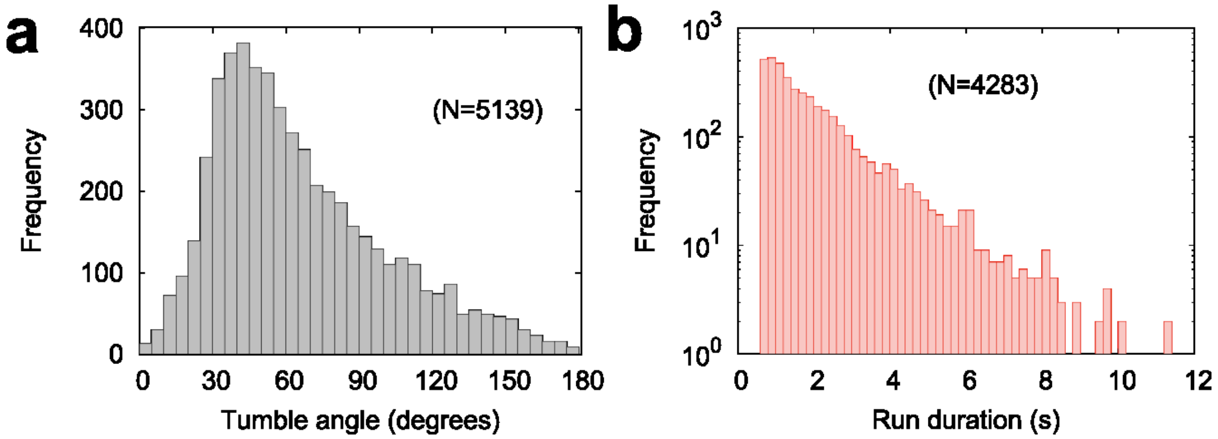

Supplementary Figure 6 Results of tracking experiments on *E. coli* strain HCB1. The swimming behaviour in this species is similar to that of the gram-positive species *Bacillus subtilis* [3], which has a chemotaxis system closely related to those in a known species of halophile archaea [4] and in our environmental strains. The data in this figure underpin the simulations of bacterial-type swimming behaviour in the main manuscript. *E. coli* cells were inoculated into lysogeny broth (LB) growth medium and incubated overnight at 30°C to saturation. 10 μl of saturated culture were used to inoculate fresh TB media for 4.5 hours at 30°C. Cells were harvested in exponential phase, washed three times into motility buffer and imaged in our DIHM setup as outlined in the methods section. Approximately 1,000 cell tracks were recorded across three biological replicates, and runs and tumbles were analysed according to the procedure previously reported [5]. The results are in close agreement with those previously reported from this species [6]. **a** Distribution of tumble angles from freely-swimming *E. coli*. Tumbles of around 45 degrees are most probable, and the mean tumble angle is 66.3 ± 0.5 degrees (the uncertainty represents standard error of the mean). **b** Distribution of run durations in freely-swimming *E. coli* strain HCB1. The distribution is exponential, with a mean run duration of 1.25 ± 0.05 s (the uncertainty represents standard error of the mean), determined by an exponential fit to the data.

Supplementary Figure 7: Simulations to show the effect of chemotactic memory length

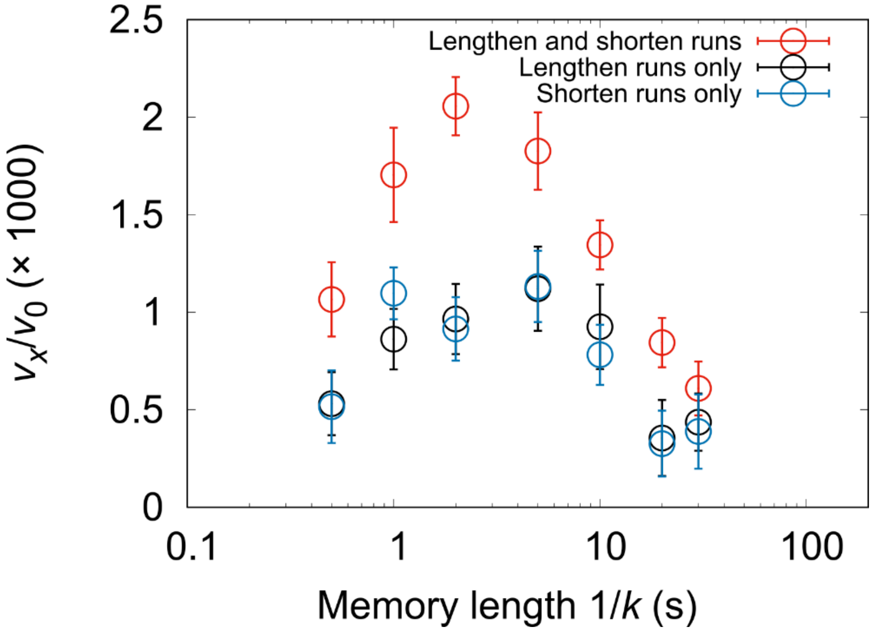

Supplementary Figure 7. Our archaea swim slowly and reverse infrequently, so rotational diffusion plays a bigger role in their swimming trajectories than in eubacteria such as *E. coli,* upon which the chemotactic model we adopt in our simulations is based. Previous modelling work has coupled the length of the chemical memory to the tumble rate (see Methods). Here, we investigate the effect of decoupling the reversal rate and the length of the memory by varying $1/k$ (loosely speaking, the 'chemotactic memory length'). Cells in these simulations had a mean run duration of 14.7 s and a swimming speed of 2 μm s^{-1}, and simulations were performed with the swimming modes indicated. The chemotactic drift velocity increases modestly with the length of the chemical memory, peaking in the region $1 < 1/k < 5$ s before decreasing with longer cell memory. This is easily understood in the case of a cell in a linear gradient such as the one we simulate: in order to compute a spatial gradient based on temporal measurements, the cell's trajectory must be approximately straight. As $1/k \to \tau_r$, the cell trajectory will meander more during the period over which the memory is averaging, reducing the usefulness of the measurement. The Brownian rotation time for our cells is approximately 6 s, consistent with a memory length of around 2 s. The rotational diffusivity of our cells is similar to that observed in experiments on *E. coli* and other bacteria [7]. Furthermore, the genetic data from our environmental isolates shows similarity to that from gram positive eubacteria, as shown in other archaeal strains [4]. This information, together with the simulation results here lead us to adopt a model for our main results section that decouples the base reversal rate from the chemotactic memory length. We have therefore chosen a chemotactic memory length of 2 s for the simulations in the main text.

Supplementary Note 1: Sampling locations – Boulby Mine and Great Salt Lake

Boulby Mine is a Permian evaporite deposit, buried approximately 1 km below the surface located in Redcar and Cleveland, UK. The deposit is a remnant of an ancient European sea, which became more saline as it dried up to form the minerals we observe today [8]. A number of halophilic archaea have been isolated from the fluid inclusion of halite crystals, which are related to modern microorganisms (e.g. references [9, 10]). Great Salt Lake is a modern hypersaline environment, and its northern arm is at salinity saturation due to the construction of a railroad causeway [11, 12]. Fluctuating with temperature effects on solubility, sodium chloride levels are measured over seasons between 24-34% [13]. The salt-saturated brine in Great Salt Lake north arm is teeming with halophilic archaea [13, 14], which respond to variations in temperature and salinity by adjusting gene regulation [15]. Though eukaryotes and bacteria also inhabit this water, halophilic archaea represent the majority of species present [16]. Studying halophilic archaea from both an ancient and modern hypersaline system provides models for a robust study of cellular structures and behaviour.

Supplementary Note 2: Helical path orientational correlation model

To improve our estimate of the archaeal cells' rotational diffusivity, we developed a simplified model for the small helical perturbation around the swimming trajectory that we observe in experiments. We assume that the rotational diffusion and helical motion are independent, and that the cell's centre of mass (the object that we track) follows a helical path around the swimming direction. The time-dependent direction vector of a non-diffusing swimmer rotating about the z-axis is given by $\mathbf{a}(t) = (\sin\theta\cos\omega t, \sin\theta\sin\omega t, \cos\theta)$. The direction correlation function is then given by the time-averaged product of two direction vectors with a delay time τ,

$$\begin{aligned} C(\tau) &= \langle \mathbf{a}(t) \cdot \mathbf{a}(t+\tau) \rangle_t \\ &= \langle \cos^2\theta + \sin^2\theta \cos(\omega t) \rangle_t, \end{aligned} \quad (1)$$

where the angle brackets denote an average over initial times t. This function is plotted in Fig. 3d. The final form is given by the product of this function with an exponential decorrelation due to rotational Brownian motion with diffusivity D_r [17]:

$$C(\tau) = exp(-2D_r\tau)[\cos^2\theta + \sin^2\theta\cos(\omega\tau)]. \quad (2)$$

Supplementary References

[1] S.D. Nuttall and M.L. Dyall-Smith, Hf1 and hf2: Novel bacteriophages of halophilic archaea, *Virology*, 197, 678–684, (1993).

[2] K. Son, F. Menolascina, and R. Stocker, Speed-dependent chemotactic precision in marine bacteria, *Proc. Natl. Acad. Sci. USA*, 113, 8624–8629, (2016).

[3] L. Turner, L. Ping, M. Neubauer, and H.C. Berg, Visualizing flagella while tracking bacteria, *Biophys. J.*, 111, 630–639, (2016).

[4] T.E.F. Quax *et al.*, and function of the archaeal response regulator CheY, *Proc. Natl. Acad. Sci. USA*, 115, E1259–E1268, (2018).

[5] M.J. Kühn *et al.*, Spatial arrangement of several flagellins within bacterial flagella improves motility in different environments, *Nat. Commun.*, 9, 5369, (2018).

[6] H. C. Berg and D. A. Brown, Chemotaxis in *Escherichia coli* analysed by three-dimensional tracking, *Nature*, 239, 500-504, (1972).

[7] G. Li and J.X. Tang, Accumulation of microswimmers near a surface mediated by collision and rotational brownian motion, *Phys. Rev. Lett.*, 103, 078101, (2009).

[8] S.H.Bottrell, M.A.Leosson, and R.J. Newton, Origin of brine inflows at Boulby potash mine, Cleveland, England, *T. I. Min. Metall. B*, 105, B159–B164, (1996).

[9] C.F. Norton, T.J. McGenity, and W.D. Grant. Archaeal halophiles (halobacteria) from two british salt mines. *J. Gen. Microbiol.*, 139, 1077–1081, (1993).

[10] T.J. McGenity, R.T. Gemmell, W.D. Grant, and H. Stan-Lotter, Origins of halophilic microorganisms in ancient salt deposits, *Env. Microbiol.*, 2, 243–250, (2000).

[11] R.J. Madison, *Effects of a causeway on the chemistry of the brine in Great Salt Lake, Utah*, Utah Geological and Mineralogical Survey, (1970).

[12] J.S. Cannon and M.A. Cannon, The southern pacific railroad trestle - past and present, in J.W. Gwynn, (ed), *Great Salt Lake: An Overview of Change*, Utah Geological Survey, (2002).

[13] S. Almeida-Dalmet, M. Sikaroodi, P.M. Gillevet, C.D. Litchfield, and B.K. Baxter, Temporal study of the microbial diversity of the north arm of Great Salt Lake, Utah, U.S., *Microorganisms*, 3, 310–326, (2015).

[14] B.L. Kemp *et al.*, The biogeography of Great Salt Lake halophilic archaea: Testing the hypothesis of avian mechanical carriers, *Diversity*, 10, 124, (2018).

[15] S. Almeida-Dalmet, C.D. Litchfield, P.M. Gillevet, and B.K. Baxter, Differential gene expression in response to salinity and temperature in a *Haloarcula* strain from Great Salt Lake, Utah, *Genes*, 9, 52, (2018).

[16] B.K. Baxter and P. Zalar, The extremophiles of Great Salt Lake: Complex microbiology in a dynamic hypersaline ecosystem, in J. Seckbach and P. Rampelotto (eds.), *Model Ecosystems in Extreme Environments*, Astrobiology Exploring Life on Earth and Beyond, Academic Press, (2019).

[17] M. Doi and S.F. Edwards. *The Theory of Polymer Dynamics*. Clarendon Press, Oxford, (1988).
